# Role of FOXO3 Activated by HIV-1 Tat in HIV-Associated Neurocognitive Disorder Neuronal Apoptosis

**DOI:** 10.3389/fnins.2019.00044

**Published:** 2019-02-04

**Authors:** Huaqian Dong, Xiang Ye, Li Zhong, Jinhong Xu, Jinhua Qiu, Jun Wang, Yiming Shao, Huiqin Xing

**Affiliations:** ^1^Cancer Research Center, Department of Basic Medical Sciences, Fujian Provincial Key Laboratory of Neurodegenerative, Disease and Aging Research, School of Medicine, Xiamen University, Xiamen, China; ^2^State Key Laboratory for Infectious Disease Prevention and Control, Collaborative Innovation Center for Diagnosis and Treatment of Infectious Diseases, National Center for AIDS/STD Control and Prevention, Chinese Center for Disease Control and Prevention, Beijing, China

**Keywords:** FOXO3, apoptosis, HIV-1 Tat, HIV-associated neurocognitive disorder, JNK

## Abstract

There are numerous types of pathological changes in human immunodeficiency virus (HIV)-associated neurocognitive disorder (HAND), including apoptosis of neurons. HIV-1 transactivator of transcription (Tat) protein, which is encoded by HIV-1, may promote apoptosis in HAND. Forkhead box O3 (FOXO3) is a multispecific transcription factor that has roles in many biological processes, including cellular apoptosis. The aim of this study was to determine whether FOXO3 is activated by HIV-1 Tat and to investigate its role in neuronal apoptosis in HAND. We employed tissue staining and related molecular biological experimental methods to confirm our hypothesis. The *in vivo* experimental results demonstrated that the expression of nuclear FOXO3 increased in the apoptotic neurons of the cerebral cortexes of rhesus macaques infected with simian human immunodeficiency virus (SHIV). The *in vitro* investigation showed that HIV-1 Tat activated FOXO3, causing it to move from the cytoplasm to the nucleus *via* the c-Jun N-terminal kinase (JNK) signaling pathway in SH-SY5Y cells. Moreover, FOXO3 down-regulated expression of the anti-apoptosis gene B-cell lymphoma 2 (Bcl-2) and up-regulated the expression of the pro-apoptosis gene Bcl-2-like 11 (Bim) after entering the nucleus, eventually causing cellular apoptosis. Finally, reduction of nuclear FOXO3 reversed cellular apoptosis. Our results suggest that HIV-1 Tat induces FOXO3 to translocate from the cytoplasm to the nucleus *via* the JNK signaling pathway, leading to neuronal apoptosis. Agents targeting FOXO3 may provide approaches for restoring neuronal function in HAND.

## Introduction

Impairment of cognition induced by human immunodeficiency virus (HIV) is known as HIV-associated neurocognitive disorder (HAND) and is an important chronic central nervous system syndrome affecting HIV-infected individuals (Antinori et al., 2007). The incidence of HAND among HIV-infected people is 15–50% (McArthur et al., 2010). Patients with HAND may suffer from cognitive dysfunction, deficits in memory and attention, and impairment of motor skills, and so on (Antinori et al., 2007).

SHIV_SF162P4_ is a chimeric simian human immunodeficiency virus, whose pathogenic features have been described (Polacino et al., 2008). This virus contains main genes from HIV-1_SF162_ (R5, macrophage-tropic [MT]/non-syncytium-inducing [NSI]) such as *tat, rev, enu*, and *vpu*, and spliced with the molecular clone simian immunodeficiency virus SIV_mac239_ (Tan et al., 1999). SHIV_SF162P4_ can cause immunological suppression and eventually cause rhesus macaques to develop acquired immune deficiency syndrome (AIDS).

The histopathological features of HAND are closely associated with neuronal injury (Fox et al., 1997), and the apoptosis of neurons has been confirmed to be related to neuronal damage (Adle-Biassette et al., 1995; Petito and Roberts, 1995). However, HIV-1 does not infect neurons directly while the neurons suffer damage (Kaul et al., 2001). HIV-1 transactivator of transcription (Tat) is one of the HIV-1-encoded proteins thought to be involved in neuronal injury caused by neurodegeneration (Cadet and Krasnova, 2007), and it can cause neuronal apoptosis both *in vitro* and *in vivo* without HIV-1 directly infecting neurons (New et al., 1997; Maragos et al., 2003). However, the apoptosis mechanism of neurons induced by HIV-1 Tat in HAND remains unknown. It is possible that HIV-1 Tat induces neuronal apoptosis via certain regulatory factors or pathways.

Forkhead box O3 (FOXO3) is a member of the FOXO transcription factor subfamily (Greer and Brunet, 2005). FOXO3 activates transcription of relevant target genes, resulting in cellular apoptosis, cell-cycle arrest, aging and DNA repair (Dijkers et al., 2000; Tran et al., 2002; Calnan and Brunet, 2008; Tia et al., 2018). Moreover, FOXO3 can be activated in HIV-1-infected macrophages to promote apoptosis of the macrophages (Cui et al., 2008). However, the involvement (or lack thereof) of FOXO3 in the neuronal apoptosis observed in HAND is still poorly understood.

Our study focused on the relationships among FOXO3, HIV-1 Tat and neuronal apoptosis in HAND. We found that expression of FOXO3 in the neuronal nucleus was elevated in HAND and that this phenomenon was associated with neuronal apoptosis in rhesus macaques infected with SHIV, which were used as HAND animal models. We hypothesized that FOXO3 is activated by HIV-1 Tat and that it participates in neuronal apoptosis in HAND.

## Materials and Methods

### SHIV-Infected Animals

Twelve rhesus macaques that were found to be seronegative for SHIV, B virus, SIV, simian T-lymphotropic virus and type D retroviruses were screened. Eight SHIV-infected rhesus macaques (#1–8) were all intravenously inoculated at the age of 280 days with SHIV_SF162P4_ (provided by Dr. Nancy Miller, National Institute of Allergy and Infectious Diseases, National Institutes of Health, Bethesda, MD, United States), and the animals were sacrificed 196 days later. Four rhesus macaques not infected with the virus (#10–13) constituted the control groups (Table 1). The rhesus macaques were kept separately in cages and maintained in rigorous accordance with the rules and guidelines of the National Institute for Infectious Diseases and Experimental Animal Welfare and the Institute of Laboratory Animal Sciences of the Chinese Academy of Medical Science (Xing et al., 2008). The protocol was approved by the Committee on the Ethics of Animal Experiments, Institute of Laboratory Animal Sciences, Chinese Academy of Medical Sciences, China. The rhesus macaques were sacrificed simultaneously after inoculation, when they showed pathological conditions as previously described (Xing et al., 2003, 2009a). On autopsy, viral loads were detected in the peripheral blood of the rhesus macaques using previously published methods (Xing et al., 2003, 2009b) (Table 1).

**Table 1:** Information about rhesus macaques used in this research.

Animal no.	Sex	Age at virus inoculation (days)	Duration of infection (days)	Age at death (days)	Viral inoculums	Viral RNA load in plasma at autopsy (copies/mL)	Clinical information	GFAP-positive cells^∗^
1	Female	280	196	476	SHIV_SF162P4_	7930000	Morbid and weight loss	2
2	Male	280	196	476	SHIV_SF162P4_	1000000	Morbid and weight loss	2
3	Female	280	196	476	SHIV_SF162P4_	1660000	Morbid and weight loss	3
4	Male	280	196	476	SHIV_SF162P4_	2290000	Morbid and weight loss	3
5	Female	280	196	476	SHIV_SF162P4_	1340000	Morbid and weight loss	2
6	Male	280	196	476	SHIV_SF162P4_	2050000	Morbid and weight loss	3
7	Female	280	196	476	SHIV_SF162P4_	4970000	Morbid and weight loss	2
8	Male	280	196	476	SHIV_SF162P4_	9650000	Morbid and weight loss	3
10	Female	/	/	1092	Control	0	/	/
11	Male	/	/	2562	Control	0	/	/
12	Male	/	/	1463	Control	0	/	/
13	Male	/	/	1771	Control	0	/	/

*^∗^Immunohistochemical staining for GFAP antigen was assessed semi-quantitatively by scoring from 0 to 3 in each animal.*

### Hematoxylin and Eosin Staining

Tissue specimens were obtained from the eight SHIV-infected rhesus macaques and the four control rhesus macaques. After being perfused with 4% paraformaldehyde, the frontal cerebral cortexes were embedded in paraffin and sliced into 5-μm thick sheets. Hematoxylin and eosin (HE) staining was performed using the Hematoxylin and Eosin Staining Kit (Beyotime Biotechnology, C0105) after dewaxing the tissue.

### Immunohistochemistry and Double-Labeling

A streptavidin peroxidase (SP) kit (Maixin Biotechnology, China, KIT-9720) was used for immunohistochemistry (IHC). Antibodies used in the IHC analysis included anti-glial fibrillary acidic protein (GFAP) antibody (1:200, Millipore, AB5541), anti-FOXO3 antibody (1:100, Abcam, ab53287), neuron specific nuclear protein (NeuN) antibody (1:200, Abcam, ab104224), cleaved caspase-3 antibody (1:200, Cell Signaling Technology, #9661). All of the experimental procedures were performed strictly in accordance with the manufacturers’ instructions.

To confirm the location of FOXO3 in rhesus macaque cerebral cortex neurons, double-labeling IHC was performed using the EnVision method. The EnVision method is also called ELPS (enhanced labeled polymer system) method. After the antigen-antibody reaction is combined, the second antibody is labeled with a multimeric compound (glucan) enzyme complex (EnVision complex), and is bound to the first antibody. Further, color development is performed by an enzyme substrate. The EnVision complex utilizes a multimeric compound to simultaneously label HRP or AKP and a secondary antibody (anti-mouse or anti-rabbit IgG) on a multimeric compound, that is to say, an enzyme-polymeric compound-second antibody macrocomplex. The secondary antibodies which we used included Simple Stain^TM^ MAX PO (R) (Nichirei, 424141) and Simple Stain^TM^ MAX AP (M) (Nichirei, 414241). FOXO3 (1:100) was labeled with 3,3’-diaminobenzidine (DAB)/peroxidase (PO) (Maixin Biotechnology, DAB0031), and then NeuN (1:200) was labeled with PermaBlue Plus/alkaline phosphatase (AP) (Diagnostic Biosystems, K058).

To confirm neuronal apoptosis in the rhesus macaques cerebral cortex and to investigate the relationship between neuronal apoptosis and the location of FOXO3 in the nucleus, we again used double-labeling IHC. Cleaved caspase-3 (1:200) was labeled with DAB/PO, and then NeuN (1:200) or FOXO3 (1:100, Proteintech, 66428-1-Ig) was labeled with PermaBlue Plus/AP.

### Cell Culture and Transfection

SH-SY5Y cell line was purchased from American Type Culture Collection (Manassas, VA, United States). The cells were maintained in basic Dulbecco’s modified Eagle’s medium (DMEM) (Gibco, C11995500BT) containing 10% fetal bovine serum (FBS) (Gibco, 12657-029), 100 mg/mL streptomycin and 100 U/mL penicillin at 37°C in a humidified incubator with 5% CO_2_.

Plasmids of pcDNA3.1-tat-flag and pcDNA3.1-flag were prepared in the *E. coli* DH5α strain and extracted by using Endofree Maxi Plasmid Kit (TIANGEN, DP117). Specific small-interfering RNA (siRNA) for FOXO3 and a scrambled negative control were purchased from Ruibo Company (Guangzhou, China). The plasmids and siRNA were transfected in the presence of reduced serum medium (Opti-MEM) (Gibco, 1440030) with Lipofectamine^®^ 2000 Transfection Reagent (Invitrogen, 11668019) in accordance with the manufacturer’s instructions.

### RNA Isolation and Quantitative Real-Time Polymerase Chain Reaction

RNA was isolated and purified from the cultured SH-SY5Y cells using TRIzol Reagent (Life Technologies, 15596018) according to the manufacturer’s instructions. Complementary DNA (cDNA) was generated by reverse transcription with the ReverTra Ace quantitative real-time polymerase chain reaction (qRT-PCR) Master Mix (Toyobo, FSQ-201), and then mix them with specific primers and FastStart Universal SYBR Green Master (ROX) (Toyobo, 11750800). qRT-PCR was performed with the AB7100 RT-PCR system (Applied Biosystems, United States). The amplification included the following process: initial incubation at 95°C°C for 10 min, 40 amplification cycles (15 s at 95°C, 1 min at 60°C), and a final melting curve from 60 to 95°C. Information regarding primers for various specific genes is presented in Table 2.

**Table 2:** Primers for the qRT-PCR Assay.

Name	Forward primer (5′–3′)	Reverse primer (5′–3′)
FOXO3	CGGACAAACGGCTCACTCT	GGACCCGCATGAATCGACTAT
Bcl-2	GGTGGGGTCATGTGTGTGG	CGGTTCAGGTACTCAGTCATCC
MCL1	GTGCCTTTGTGGCTAAACACT	AGTCCCGTTTTGTCCTTACGA
P53	TGTGACTTGCACGTACTCCC	ACCATCGCTATCTGAGCAGC
Bim	CAGTGCAATGGCTTCCATGAG	GTATCTCGGCTCCGCAAAGA
Trail	CTGAAGCAGATGCAGGACAAGT	TGCTACTCTCTGAGGACCTCTTT
FasL	CTACCAGCCAGATGCACACA	CCTTGAGTTGGACTTGCCTGT
β-actin	CATGTACGTTGCTATCCAGGC	CTCCTTAATGTCACGCACGAT

### Western Blotting Analysis

Whole-cell lysates of SH-SY5Y cells were obtained using radio immunoprecipitation assay (RIPA) lysis buffer (Boster Biological Technology, AR0105-100) containing protease inhibitor (Roche, 04693132001) and phosphatase inhibitor (Roche, 04906845001). Nuclear and cytoplasmic proteins of SH-SY5Y cells were, respectively, obtained by using the NE-PER^®^ Nuclear and Cytoplasmic Extraction Reagents (Thermo Scientific, #78833). The concentration of protein was measured using a bicinchoninic acid assay (BCA) Protein Assay Kit (Thermo Scientific Pierce, 23228). Cell lysates (20 μg) were loaded and separated with sodium dodecyl sulfate-polyacrylamide gel electrophoreses (SDS-PAGE) (10–15% polyacrylamide gel) and were transferred onto Immobilon PVDF Transfer Membrane (Sigma, IPVH00010) followed by blocking with 5% skim milk powder in phosphate-buffered saline (PBS) containing 0.1% Tween20 (PBS-T) at room temperature for 1 h. Afterward, the membrane were incubated with appropriate primary antibodies, including HIV-1 Tat (1:1000, Abcam, ab63957), GAPDH (1:10000, Abcam, ab181602), FOXO3 (1:1000, Abcam, ab53287), Phospho-FOXO3(S253) (1:1000, Abcam, ab47285), c-Jun (1:1000, Cell Signaling Technology, #9165), Phospho-c-Jun (1:1000, Abcam, ab4821), AKT (1:1000, Cell Signaling Technology, #9272), Phospho-Akt (Ser473) (1:2000, Cell Signaling Technology, #4060), Bim (1:1000, Abcam, ab32158), Mcl-1 (1:1000, Abcam, ab32087), Bcl-2 (1:1000, Abcam, ab32124), Trail (1:1000, Cell Signaling Technology, #3219) and P53 (1:200, Santa Cruz Biotechnology, sc-126) for one night at 4°C atmosphere. The membrane was then incubated with horse-radish peroxidase (HRP)-labeled secondary antibodies (1:3000, Thermo Fisher, 62-6520 and R&D Systems, HAF008) for 1 h at room temperature. Signals of the protein bands were detected with Immobilon Western Chemiluminescent HRP Substrate (Millipore, WBKLS0500).

### Immunofluorescence

For immunofluorescence of FOXO3, SH-SY5Y cells were seeded on glass coverslips in 24-well plates and transfected pcDNA3.1-tat-flag or pcDNA3.1-flag plasmid for 48 h. The cells were then fixed for 30 min in 4% paraformaldehyde solution in PBS at room temperature, permeabilized in 0.5% Triton X-100 in PBS for 10 min and blocked with blocking buffer [5% bovine serum albumin (BSA)]. Cells were then incubated with anti-FOXO3 antibody (1:50, Abcam) at 4°C overnight. Cells were then washed four times with 1 × PBS and incubated on coverslips with fluorescent secondary antibodies (Thermo, #31506) at room temperature for 1 h. For nuclear staining, the cells were stained with 4’,6-diamidino-2-phenylindole (DAPI) (VECTOR, H-1200).

### Terminal Deoxynucleotidyl Transferase dUTP Nick End Labeling Assay

Prior to the experiment, SH-SY5Y cells were seeded in 24-well plates and treated with plasmids for 48 h. The cells were then fixed in freshly prepared 4% formaldehyde in PBS for 25 min at 4°C. After washing the slides with 1 × PBS, cells were permeabilized in 0.2% Triton X-100 solution in PBS for 5 min. After rinsing the sheets with PBS, cells were stained with the DeadEND^®^ Fluorometric TUNEL System (Promega, G3250) in accordance with the manufacturer’s instructions.

### Measurement of Caspase 3/7 Activity

First, the SH-SY5Y cells were inoculated into a 96-well plate and then transfected with plasmid for 48 h. After the cells were processed, the 96-well plate containing cells was removed from the incubator and equilibrated to room temperature. The caspase 3/7 activity was then determined using Caspase-Glo^®^ 3/7 Assay Systems (Promega, G8090) according to the operating instructions.

### Chemicals

SP600125 (Abcam, ab120065), diallyl tetrasulfide (DSA4) (Abcam, ab143603), staurosporine (STS) (Cell Signaling Technology, #9953), and LY294002 (Millipore, #19–142) were solubilized in dimethyl sulfoxide (DMSO) before use.

### Statistical Analysis

All results were derived from at least three independent repeated experiments. The results were expressed as mean ± standard deviation. One-way analysis of variance and unpaired-samples *t*-test were used to analyze the data with GraphPad Prism 5.0 statistical software. The level of significance was set at *P* < 0.05.

## Results

### Viral RNA Loads of the SHIV-Infected Rhesus Macaques

Eight rhesus macaques (#1–8) were infected with SHIV, and four (#10–13) uninfected macaques were used as controls (Table 1). Viral RNA loads in the peripheral blood of the rhesus macaques were analyzed on autopsy. As shown in Table 1, all of the SHIV-infected rhesus macaques showed high viral loads and the uninfected rhesus macaques showed no viral loads.

### Pathological Changes in the SHIV-Infected Rhesus Macaque Cerebral Cortex

**FIGURE 1:**
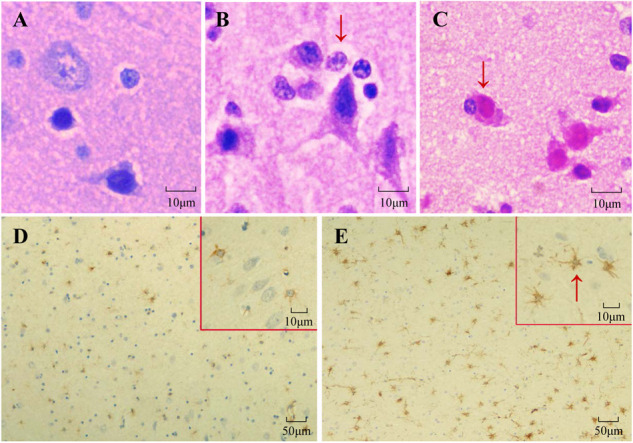
Histopathologic examination of rhesus macaque cerebral cortexes. **(A–C)** HE staining of rhesus macaque cerebral cortexes. **(A)** Normal cerebral cortex of the control group. **(B)** Satellitosis of SHIV-infected rhesus macaques. **(C)** Neuronophagia of SHIV-infected rhesus macaques. **(D,E)** The accumulation of GFAP and the quantity of astrocytes were increased in the cerebral cortex of the SHIV-infected rhesus macaques **(E)** compared with the control group **(D)**, as shown by GFAP (brown) using immunohistochemistry.

HE staining and IHC staining were used to reveal the pathological changes in the cerebral cortexes of SHIV-infected rhesus macaques. Satellitosis and neuronophagia were observed in the cerebral cortexes of the SHIV-infected rhesus macaques (Figure 1A–C). Diffuse astrocytic gliosis and increased accumulation of the intermediate filament GFAP were detected in the cerebral cortexes of SHIV-infected rhesus macaques (Figure 1D,E). There was no correlation between different viral RNA loads in plasma and GFAP-positive cells in the cerebral cortex of SHIV-infected rhesus macaques (Table 1). These results indicated that the pathological manifestations of the SHIV-infected rhesus macaques cerebral cortex was very similar to those in the cerebral cortex of HAND patients (Navia et al., 1986; Masliah et al., 1992; Fan and He, 2016).

### FOXO3 Is Increased in the Neuronal Nucleus and It Is Associated With the Neuronal Apoptosis in the SHIV-Infected Rhesus Macaque Cerebral Cortex

**FIGURE 2:**
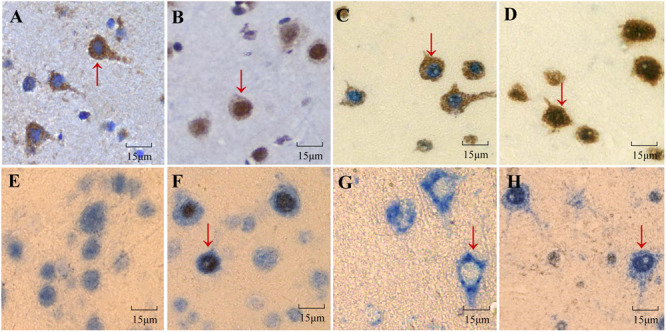
Expression of FOXO3 in rhesus macaque cerebral cortexes and its correlation with neuronal apoptosis. **(A,B)** Expression of FOXO3 (brown) in the nuclei of cells of the cerebral cortexes of SHIV-infected rhesus macaques **(B)** was higher than that of the control group **(A)**, as analyzed by immunohistochemistry. **(C,D)** Double-labeled IHC demonstrated that nuclear FOXO3 expression was higher in the nuclei of neurons of the cerebral cortexes of SHIV-infected rhesus macaques **(D)** than in the control group **(C)** (FOXO3 was brown, NeuN was blue). **(E,F)** Neuronal apoptosis in the cerebral cortex of SHIV-infected rhesus macaques **(F)** was manifested by cleaved caspase-3 (brown) and NeuN (blue) through double-labeled IHC in contrast with little neuronal apoptosis of the control group **(E)**. **(G,H)** The expression of nuclear FOXO3 in apoptotic cells of SHIV-infected rhesus macaques was higher than that in the control group according to FOXO3 (blue) and cleaved caspase-3 (brown) using double-labeled IHC.

The expression of FOXO3 in the cellular nuclei of SHIV-infected rhesus macaques cerebral cortexes was higher than that of the control group, as determined by IHC (Figure 2A,B). In addition, we performed double-labeled IHC for FOXO3 in combination with NeuN. Those results indicated that the FOXO3 neuronal nucleus expression in the cerebral cortexes of the SHIV-infected group was higher than that of the control group (Figure 2C,D). We then utilized double-labeled IHC for cleaved caspase-3 and NeuN to reveal neuronal apoptosis in the cerebral cortex. There was little neuronal apoptosis in the uninfected rhesus macaque cerebral cortexes, as opposed to more neuronal apoptosis in the SHIV-infected group (Figure 2E,F). Additionally, to further investigate the relationship between increased nuclear expression of FOXO3 and neuronal apoptosis, double-labeled IHC for FOXO3 and cleaved caspase-3 was performed. Neurons were judged based on the shape of cells and the size of the nuclei. There was an increase of FOXO3 in the nuclei of apoptotic neurons (Figure 2G,H). These results revealed that neurons with increased nuclear expression FOXO3 were prone to apoptosis in the SHIV-infected rhesus macaque cerebral cortexes.

### HIV-1 Tat Activates FOXO3 to Transfer From Cytoplasm to Nucleus in SH-SY5Y Cells

**FIGURE 3:**
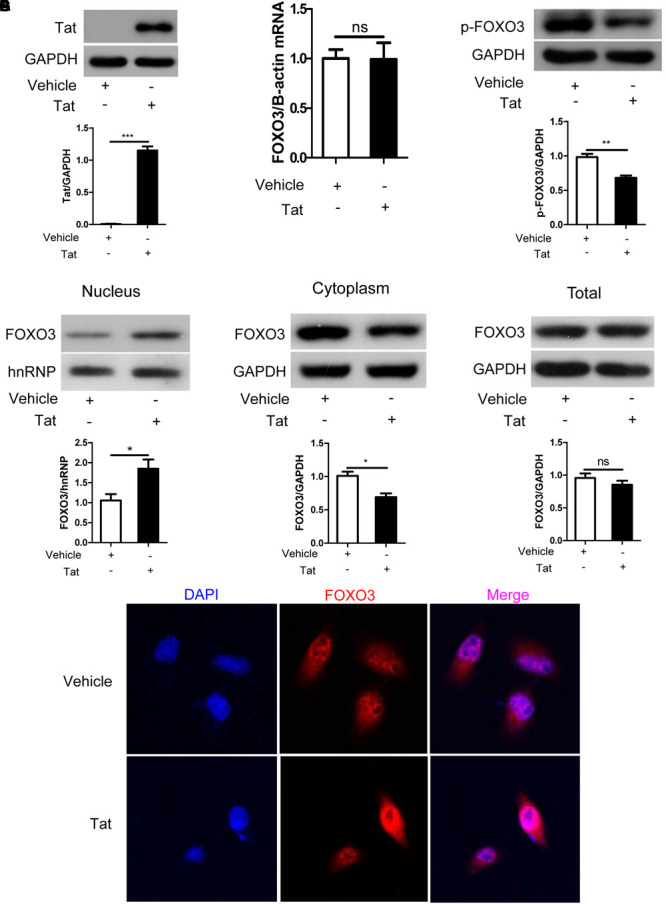
HIV-1 Tat alters FOXO3 intracellular localization in SH-SY5Y cells. SH-SY5Y cells were transfected with pcDNA3.1-flag (4 μg) or pcDNA3.1-Tat-flag (4 μg) for 48 h. **(A)** Expression of HIV-1 Tat was measured with Western blotting. Statistical analysis is below (^∗∗∗^*P* < 0.001) **(B)** Statistical analysis of the mRNA level of FOXO3 as measured by qRT-PCR. **(C)** The expression of phosphorylated FOXO3 was analyzed with Western blotting. Statistical analysis is below (^∗∗^*P* < 0.01). **(D–F)** The expression of nuclear FOXO3, cytoplasmic FOXO3 and total FOXO3 was analyzed by Western blotting. Statistical analysis is below (^∗^*P* < 0.05). **(G)** The nuclear translocation of FOXO3 was observed by confocal microscopy using immunofluorescence staining. Red indicates FOXO3 and blue indicates nuclei. Pictures were magnified 400×. Statistical analyses of all Western blots were of at least three independent repeated experiments. Vehicle: pcDNA3.1-flag; Tat: pcDNA3.1-Tat-flag.

To investigate the relationship between HIV-1 Tat and FOXO3 at the cellular level, we chose SH-SY5Y cells and transfected them with plasmids pcDNA3.1-Tat-flag or pcDNA3.1-flag for 48 h. Expression of HIV-1 Tat was measured by Western blotting, HIV-1 Tat was only expressed in cells transfected with pcDNA3.1-Tat-flag (Figure 3A). We then used the two plasmids aforementioned to transfect SH-SY5Y cells separately in order to detect FOXO3 changes in mRNA and protein levels. There was no significant change in FOXO3 at the mRNA level (Figure 3B). However, we found that HIV-1 Tat significantly reduced the expression of phosphorylated FOXO3 (Figure 3C). Decreased phosphorylation of FOXO3 is equivalent to increased levels of dephosphorylation of FOXO3, which indicates that it FOXO3 is activated, which causes it to enter the nucleus to play a role in promoting transcription (Brunet et al., 1999). We then extracted the cytoplasmic and nuclear proteins from the SH-SY5Y cells and quantified FOXO3 expression separately in the cytoplasm and nucleus. At the same time, we detected the protein expression of FOXO3 in SH-SY5Y whole-cell lysate. We found that HIV-1 Tat increased the expression of FOXO3 in the nucleus and decreased the expression of FOXO3 in the cytoplasm, but had no significant effect on the expression of total FOXO3 (Figure 3D–F). Immunofluorescence confirmed the result (Figure 3G). These results indicated that HIV-1 Tat activated FOXO3 to translocate into the nuclei of SH-SY5Y cells.

### HIV-1 Tat Regulation of FOXO3 Intracellular Localization *via* JNK Signaling Pathway in SH-SY5Y Cells

**FIGURE 4:**
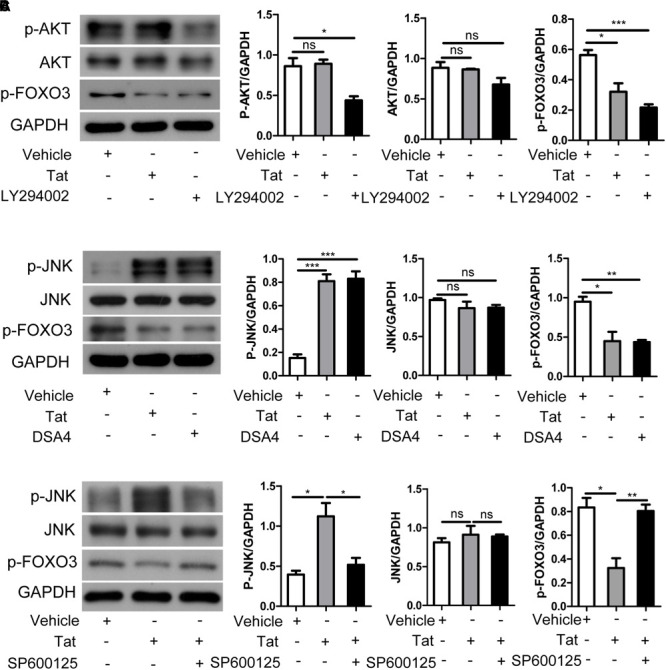
HIV-1 Tat regulates FOXO3 *via* the JNK signaling pathway. **(A)** SH-SY5Y cells were transfected with pcDNA3.1-flag or pcDNA3.1-Tat-flag for 48 h or treated with PI3K-Akt inhibitor LY294002 (20 μg/mL). The expression of phosphorylated AKT, AKT and phosphorylated FOXO3 was analyzed by Western blotting. Statistical analysis is on the right (^∗^*P* < 0.05). **(B)** SH-SY5Y cells were transfected with pcDNA3.1-flag or pcDNA3.1-Tat-flag for 48 h or treated with JNK activator DSA4 (4 μM). The expression of phosphorylated JNK, JNK and phosphorylated FOXO3 was analyzed by Western blotting. Statistical analysis is on the right (^∗^*P* < 0.05; ^∗∗^*P* < 0.01; ^∗∗∗^*P* < 0.001). **(C)** SH-SY5Y cells were transfected with pcDNA3.1-flag or pcDNA3.1-Tat-flag only for 48 h, or were pre-treated with the JNK inhibitor SP600125 (20 μM) followed transfection with pcDNA3.1-Tat-flag for 48 h. The expression of phosphorylated JNK, JNK and phosphorylated FOXO3 was analyzed by Western blotting. Statistical analysis is on the right (^∗^*P* < 0.05; ^∗∗^*P* < 0.01). Statistical analyses of all Western blots were of at least three independent repeated experiments. Vehicle: pcDNA3.1-flag; Tat: pcDNA3.1-Tat-flag.

To investigate how HIV-1 Tat regulates FOXO3 intracellular localization, we transfected SH-SY5Y cells with plasmids pcDNA3.1-Tat-flag or pcDNA3.1-flag, or treated SH-SY5Y cells with DSA4 (a specific JNK activator), or treated them with the AKT signaling pathway inhibitor LY294002. We found that HIV-1 Tat had no significant effect on the expression and existential forms of AKT, but the phosphorylation level of FOXO3 was decreased by inhibiting AKT with LY294002 (Figure 4A). HIV-1 Tat was found to induce phosphorylation of JNK but had no effect on total JNK, which was similar to DSA4 (Figure 4B). At the same time, HIV-1 Tat promoted dephosphorylation of FOXO3, as did DSA4. To further validate the role of the JNK signaling pathway in HIV-1 Tat regulating the change in position of FOXO3 within the cell, we utilized plasmids pcDNA3.1-Tat-flag or pcDNA3.1-flag to transfect SH-SY5Y cells, or we treated SH-SY5Y cells with the JNK inhibitor SP600125 followed by transfection with pcDNA3.1-Tat-flag. We found that HIV-1 Tat promoted phosphorylation of JNK but had no influence on the total expression of JNK and that it facilitated dephosphorylation of FOXO3 (Figure 4C). This effect of HIV-1 Tat was impaired when JNK was inhibited. These results demonstrated that HIV-1 Tat promoted dephosphorylation of FOXO3 to migrate it from cytoplasm to nucleus via the JNK signaling pathway in SH-SY5Y cells.

### Activated FOXO3 Induction of Apoptosis by Regulating Downstream Pro-apoptotic/Anti-apoptotic Genes Expression

**FIGURE 5:**
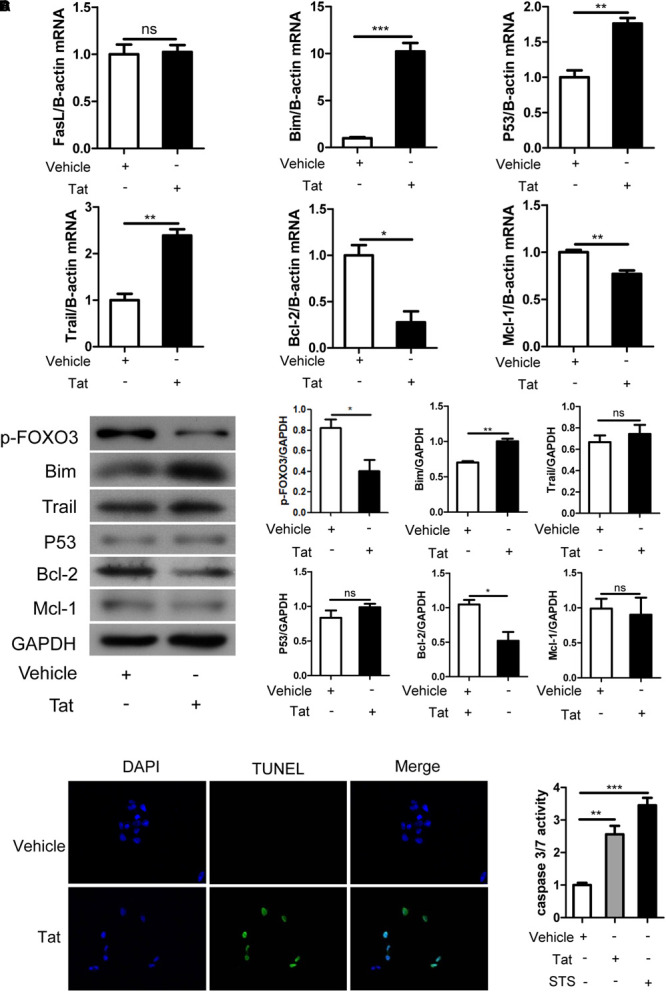
Activated FOXO3 induces apoptosis by regulating downstream apoptosis-related genes. SH-SY5Y cells were transfected with pcDNA3.1-flag or pcDNA3.1-Tat-flag for 48 h. **(A)** Statistical analysis of the mRNA level of pro-apoptotic gene FasL, Bim, P53, Trail and anti-apoptotic gene Bcl-2, Mcl-1 measured by qRT-PCR. **(B)** The expression of Bim, Trail, P53, Bcl-2 and Mcl-1 was analyzed by Western blotting. Statistical analysis is on the right (^∗^*P* < 0.05; ^∗∗^*P* < 0.01). **(C)** The apoptosis of SH-SY5Y cells was visualized with TUNEL staining and observed by confocal microscopy. Green indicates apoptosis cells and blue indicates cellular nuclei. Pictures were magnified 400×. **(D)** The cells were transfected with plasmids or treated with STS (0.5 μM) for 48 h. The apoptosis of SH-SY5Y cells was detected by measuring caspase 3/7 activity, and the statistical analysis is presented (^∗∗^*P* < 0.01; ^∗∗∗^*P* < 0.001). Statistical analyses of all Western blotting were of at least three independent repeated experiments. Vehicle: pcDNA3.1-flag; Tat: pcDNA3.1-Tat-flag.

We next explored whether apoptosis-related gene expression and cellular apoptosis would be affected after the activation of FOXO3 induced by HIV-1 Tat. SH-SY5Y cells were transfected with pcDNA3.1-Tat-flag or pcDNA3.1-flag in order to active FOXO3, and expression of pro-apoptotic/anti-apoptotic genes was measured by qRT-PCR and Western blotting. At the transcriptional level, activated FOXO3 caused a significant increase in the transcription of pro-apoptotic genes Bim, Trail and P53, but no obvious change in Fas Ligand (FasL) transcription, and activated FOXO3 caused a distinct decrease in the transcriptional level of the anti-apoptotic genes BCL2 and MCL1 (Figure 5A). We then investigated whether the gene changes induced by activated FOXO3 at the transcriptional level were also altered at the protein level. As at the transcription levels, activated FOXO3 caused an increase in Bim protein expression and a decrease in Bcl-2 protein expression. However, we did not find any apparent changes in the protein level of Trail, P53, or Mcl-1 (Figure 5B).

To confirm the relationship between the activated FOXO3 induced by HIV-1 Tat and cellular apoptosis, SH-SY5Y cells were transfected with pcDNA3.1-Tat-flag or pcDNA3.1-flag for 48 h and then assessed by TUNEL assay. As shown in Figure 5C, activated FOXO3 caused a significant increase in TUNEL positivity. We also transfected the SH-SY5Y cells separately with the plasmids aforementioned for 48 h or treated them with STS (which caused cellular apoptosis) for 48 h; we then analyzed caspase 3/7 activity. As shown in Figure 5D, activated FOXO3 gave rise to a remarkable increase in caspase 3/7 activity. These data indicated that FOXO3 was activated by HIV-1 Tat and in turn selectively regulated the changes of its downstream pro-apoptotic and anti-apoptotic genes, eventually causing the apoptosis of SH-SY5Y cells.

### FOXO3 Plays a Key Role in Inducing Neuronal Apoptosis

**FIGURE 6:**
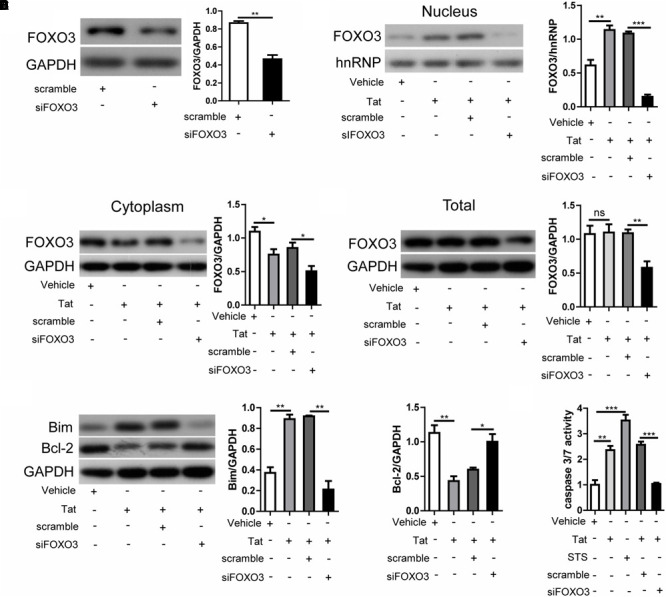
FOXO3 plays a vital role in neuronal apoptosis. SH-SY5Y cells were transfected with pcDNA3.1-flag or pcDNA3.1-Tat-flag only for 48 h, or were treated with scramble-siRNA or FOXO3-siRNA for 24 h followed by transfection with pcDNA3.1-Tat-flag for 48 h. **(A)** Expression of FOXO3 was analyzed by Western blotting. Statistical analysis is on the right (^∗∗^*P* < 0.01). **(B)** Expression of nuclear FOXO3 was analyzed by Western blotting. Statistical analysis is on the right (^∗∗^*P* < 0.01; ^∗∗∗^*P* < 0.001). **(C)** Expression of cytoplasmic FOXO3 was analyzed by Western blotting. Statistical analysis is on the right (^∗^*P* < 0.05). **(D)** Expression of total FOXO3 was analyzed by Western blotting. Statistical analysis is on the right (^∗∗^*P* < 0.01). **(E)** Expression of Bim and Bcl-2 was analyzed by Western blotting. Statistical analysis is on the right (^∗∗^*P* < 0.01). **(F)** Apoptosis of SH-SY5Y cells was detected by measuring caspase 3/7 activity, and the statistical analysis is presented (^∗∗^*P* < 0.01; ^∗∗∗^*P* < 0.001). Statistical analyses of all Western blots were of at least three independent repeated experiments. Vehicle: pcDNA3.1-flag; Tat: pcDNA3.1-Tat-flag. Scramble: scramble-siRNA. siFOXO3: FOXO3-siRNA.

To further investigate the role of FOXO3 in cellular apoptosis, we transfected SH-SY5Y cells with only pcDNA3.1-Tat-flag or pcDNA3.1-flag, or transfected SH-SY5Y cells with scramble-siRNA or FOXO3-siRNA before pcDNA3.1-Tat-flag. Treatment with FOXO3-siRNA alone significantly reduced the expression of FOXO3 over that observed with transfection with scramble-siRNA (Figure 6A). In nuclear protein, knockdown of FOXO3 visibly reduced the increase in FOXO3 nucleoprotein caused by HIV-1 Tat (Figure 6B). Both the cytoplasmic FOXO3 and total FOXO3 were lower with knocking down FOXO3 before treatment with pcDNA3.1-Tat-flag than when treated with pcDNA3.1-Tat-flag and scramble-siRNA (Figure 6C,D). In addition, the increase in Bim and the decrease in Bcl-2 were not observed when FOXO3 was suppressed (Figure 6E). Similar issues were observed in the caspase 3/7 activity experiment (Figure 6F). These results indicated that FOXO3 was the key event required for apoptosis of SH-SY5Y cells.

## Discussion

Although neuronal apoptosis is a significant pathological characteristic of HAND (Adle-Biassette et al., 1995; Petito and Roberts, 1995), the mechanism remains unclear. Additionally, there have been reports that FOXO3 is associated with cellular apoptosis and that its active form may facilitate apoptosis of macrophages infected by HIV-1 (Calnan and Brunet, 2008; Cui et al., 2008). Nevertheless, the role of FOXO3 in HAND has not been fully addressed. Therefore, this study aimed to elucidate whether the transcriptional regulator protein FOXO3 regulates neuronal damage of HAND.

We first chose SHIV-infected rhesus macaques as animal models for research. The rhesus macaque is one of the most-frequently utilized animal models for HIV-associated pathogenesis research because it tends to develop AIDS-like diseases caused by primate lentivirus infection (Hu, 2005; Lackner and Veazey, 2007; Polacino et al., 2008). SHIV is an infectious recombinant virus between HIV-1 and SIVmac239 (first isolated from rhesus macaques with AIDS-like diseases or lymphoma); it can establish sustained infection in rhesus macaques and generates symptoms similar to those induced by HIV-1 in humans, including lag in response and bradykinesia, which are the clinical features of HAND (Luciw et al., 1995; Lu et al., 1996; Vlasak and Ruprecht, 2006; Antinori et al., 2007; Polacino et al., 2008). Here, we chose the cerebral cortex of rhesus macaques for detection because this brain region is known to be home to many of the HIV-1-associated neurocognitive deficits in HAND patients (Lopez-Villegas et al., 1997). Moreover, satellitosis and neuronophagia were observed with HE staining in our research. Satellitosis and neuronophagia are phenomena that have been proven to occur in neurodegenerative diseases and the important pathological change of them are neuronal death (Troost et al., 1993; Sarnat and Flores-Sarnat, 2013), characteristics that are consistent with HAND. Furthermore, IHC revealed activation of astrocytes, characterized by diffuse astrocytic gliosis and increased cytoplasmic accumulation of the intermediate filament GFAP, in the cerebral cortexes of SHIV-infected rhesus macaques in our study, which is the histopathologic hallmark of HAND (Navia et al., 1986; Masliah et al., 1992; Fan and He, 2016). Thus, SHIV-infected rhesus macaques were considered a good animal model for studying HAND.

It has been reported that an increase in FOXO3 activity causes neurodegenerative diseases such as Alzheimer’s and Parkinson’s (Qin et al., 2008; Su et al., 2009). According to others previous research (Calnan and Brunet, 2008), FOXO3 may regulate the transcriptional expression of apoptosis-related genes and, thereby, regulate cellular apoptosis (Birkenkamp and Coffer, 2003), which is involved in the neuronal pathological changes of HAND. Furthermore, neurons are the most abundant cell type in the cerebral cortex; therefore, FOXO3’s effects on neurons may have significant disease implications. We investigated whether the expression of FOXO3 had some changes in the neurons of SHIV-infected rhesus macaque cerebral cortexes. Our results showed that FOXO3 was widely expressed in the cerebral cortex and that the increase in nuclear FOXO3 may be associated with neuronal apoptosis in SHIV-infected rhesus macaques.

How is the expression of FOXO3 regulated in neurons? Recent studies have revealed that neurons are damaged despite the fact that HIV-1 does not directly infect them (Kaul et al., 2001). Some studies have indicated that several HIV-1-encoded proteins are primary causes of neuronal injury in HAND (Kaul et al., 2001). HIV-1 Tat, a potent neurotoxic viral protein (Nath et al., 1996; Chang et al., 2011) secreted by HIV-infected cells such as glial cells (Tardieu et al., 1992; Ensoli et al., 1993), is one of the most widely detected HIV-1-encoded proteins in the serum and cerebrospinal fluid of HIV-1-infected patients and is thought to be a crucial mediator of neurological dysfunction (Nath, 2002). Additionally, experiments performed in transgenic mice that specifically express HIV-1 Tat protein, have confirmed that transgenic mice without HIV-1 infection but expressing HIV-1 Tat exhibit similar recognition and behavioral changes as HAND patients (Kim et al., 2003). Furthermore, our former works have demonstrated that HIV-1 Tat may participate in the pathogenesis of HAND (Ye et al., 2017; Wu et al., 2018). Accordingly, we surmised that the expression of FOXO3 in neurons potentially regulated by HIV-1 Tat in HAND. We chose SH-SY5Y for the further cellular level exploration because it is a human-derived neuroblastoma cell line that is frequently used as a model for researching neurodegenerative disease such as Alzheimer’s and Parkinson’s (Krishna et al., 2014). Research has found that the transcriptional activity of FOXO3 is regulated by various mechanisms, including ubiquitination, acetylation, and phosphorylation (Calnan and Brunet, 2008). In some studies related to cellular apoptosis, expression or activation of FOXO3 favored the phosphorylated phenotype; researchers, also showed that inactivating phosphorylation of FOXO3 resulted in its export from the nucleus and inhibition of its transcription of pro-apoptotic genes (Brunet et al., 1999; Modur et al., 2002; You et al., 2006; Rosich et al., 2013). Interestingly, our results indicated that HIV-1 Tat could reduce the phosphorylation of FOXO3 and facilitate the dephosphorylation of FOXO3 in SH-SY5Y cells. Furthermore the nucleus and cytoplasm separation assay demonstrated that FOXO3 was dephosphorylated by HIV-1 Tat to be an activated status and then translocate from the cytoplasm to nucleus.

There are two primary mechanisms for regulation of FOXO3: phosphoinositide 3-kinase/AKT pathway and the JNK pathway (Huang and Tindall, 2007). AKT can phosphorylate FOXO3 and leads to FOXO3 sequestration in the cytoplasm (Brunet et al., 1999); conversely, JNK promotes dephosphorylating of FOXO3 and translocation into the nucleus (Li et al., 2015). Therefore, we aimed to address whether these two pathways were involved in the regulation of FOXO3 induced by HIV-1 Tat in SH-SY5Y cells. Our results illustrated that HIV-1 Tat caused activation of JNK but had no effect on AKT. The role of JNK in the HIV-1 Tat-induced dephosphorylating of FOXO3 is further demonstrated by our finding that inhibiting JNK remitted the HIV-1 Tat-induced reducing in phosphorylated FOXO3. As a result, we concluded that HIV-1 Tat activated FOXO3 through the JNK signaling pathway rather than through the AKT signaling pathway.

In accordance with the departed survey, dephosphorylated FOXO3 translocated to the nucleus and became transcriptionally active to participate in regulating apoptosis, cell cycle, DNA repair and so on (Brunet et al., 1999; Calnan and Brunet, 2008; Tia et al., 2018). Results of the tissue experiments suggested that increased nuclear FOXO3 might be involved in the neuronal apoptotic process of HAND. FOXO3 may target downstream transcriptional pathways by activating or deactivating apoptosis-related genes; the most-studied of these include the pro-apoptotic genes FasL, Bim, P53, and Trail, and the anti-apoptotic gene BCL2, MCL1 (Brunet et al., 1999; Modur et al., 2002; You et al., 2006; Rosich et al., 2013). Disequilibrium of Bim and Bcl-2 or Mcl-1 results in disruption of mitochondrial transmembrane potential, facilitating activation of caspases and eventually leading to cellular apoptosis (Schmidt et al., 2006; Krishna et al., 2011; Anilkumar and Prehn, 2014). FasL and Trail can bind to their receptors in turn causing caspase cascades and propagating the process of cellular apoptosis (Nagata, 1997; Vasaikar et al., 2015). The P53 protein regulates cellular apoptosis through multiple pathways, including activating the expression of pro-apoptotic genes and promoting the formation of apoptosome (Wawryk-Gawda et al., 2014; Deng et al., 2017). To identify genes incorporated in our model, we utilized qRT-PCR to define that Bim, P53, Trial, Bcl-2 and Mcl-1—but not FasL— as novel targets regulated by nuclear FOXO3 at the transcriptional level. Although we uniformly observed that nuclear accessorial FOXO3 increased Bim and decreased Bcl-2 at the mRNA, as well as the protein levels, we were unable to find changes in P53, Trail, or Mcl-1 at the protein level; these results were inconsistent with their mRNA levels. Using the TUNEL assay and analysing the activity of caspase 3/7, our results proved that activated FOXO3 induced by HIV-1 Tat triggered the apoptosis of SH-SY5Y cells by regulating the expression Bim and Bcl-2. To further confirm the role of FOXO3 in neuronal apoptosis, we found that when the FOXO3 was knocked down, FOXO3 entering the nucleus was consequently reduced. Meanwhile, silencing FOXO3 restored the expression of Bim and Bcl-2, and altered the SH-SY5Y cellular apoptosis. On the basis of these results we concluded that FOXO3 had a vital effect on the apoptosis of SH-SY5Y cells.

In summary, our work reveals for the first time that FOXO3 can be activated by HIV-1 Tat *via* the JNK signaling pathway and then promotes neuronal apoptosis by means of direct transcriptional repression of Bcl-2 and activation of Bim. We considered that FOXO3-dependent neural apoptosis may play a role in the pathogenesis of HAND. Hence, we provide here a further understanding of the HAND pathogenesis mechanism and the FOXO3 may be the potential promising target for the therapy of HAND.

## Author Contributions

HX instructed all investigations. HD designed and performed the experiments, collected and analyzed the data, and wrote and edited the manuscript. XY designed the research and reviewed the manuscript. LZ revised the manuscript. JX, JQ, and JW prepared the materials. YS provided SHIV-infected rhesus macaque specimens.

## Conflict of Interest Statement

The authors declare that the research was conducted in the absence of any commercial or financial relationships that could be construed as a potential conflict of interest.
