# Corrigendum: Role of FOXO3 Activated by HIV-1 Tat in HIV-Associated Neurocognitive Disorder Neuronal Apoptosis

**DOI:** 10.3389/fnins.2021.825158

**Published:** 2022-01-10

**Authors:** Huaqian Dong, Xiang Ye, Li Zhong, Jinhong Xu, Jinhua Qiu, Jun Wang, Yiming Shao, Huiqin Xing

**Affiliations:** ^1^Cancer Research Center, Department of Basic Medical Sciences, Fujian Provincial Key Laboratory of Neurodegenerative, Disease and Aging Research, School of Medicine, Xiamen University, Xiamen, China; ^2^State Key Laboratory for Infectious Disease Prevention and Control, Collaborative Innovation Center for Diagnosis and Treatment of Infectious Diseases, National Center for AIDS/STD Control and Prevention, Chinese Center for Disease Control and Prevention, Beijing, China

**Keywords:** FOXO3, apoptosis, HIV-1 Tat, HIV-associated neurocognitive disorder, JNK

There is an error in the Funding statement. The correct number for the Natural Science Foundation of Fujian Province of China is No. 2018J01134.

The authors apologize for this error and state that this does not change the scientific conclusions of the article in any way. The original article has been updated.

Incorrect Affiliation.

In the published article, there was an error in affiliation 1. Instead of “Fujian Provincial Key Laboratory of Neurodegenerative Disease and Aging Research, Institute of Neuroscience, Department of Pathology, Basic Medicine, Medical College, Xiamen University, Xiamen, China”, it should be “Cancer Research Center, Department of Basic Medical Sciences, Fujian Provincial Key Laboratory of Neurodegenerative, Disease and Aging Research, School of Medicine, Xiamen University, Xiamen, China”.

## Publisher's Note

All claims expressed in this article are solely those of the authors and do not necessarily represent those of their affiliated organizations, or those of the publisher, the editors and the reviewers. Any product that may be evaluated in this article, or claim that may be made by its manufacturer, is not guaranteed or endorsed by the publisher.

